# Blood Pressure is Associated With Cerebral Blood Flow Alterations in Patients With T2DM as Revealed by Perfusion Functional MRI

**DOI:** 10.1097/MD.0000000000002231

**Published:** 2015-12-07

**Authors:** Wenqing Xia, Hengyi Rao, Andrea M. Spaeth, Rong Huang, Sai Tian, Rongrong Cai, Jie Sun, Shaohua Wang

**Affiliations:** From the Department of Endocrinology, Affiliated Zhongda Hospital of Southeast University, No. 87 Dingjiaqiao Road, Nanjing (WX, RH, ST, RC, JS, SW); Medical School of Southeast University, No.87 Dingjiaqiao Road, Nanjing, China (WX, SW); Center for functional Neuroimaging, University of Pennsylvania, 3710 Hamilton Walk, Philadelphia, PA (WX, HR); and Center for Sleep and Circadian Neurobiology, Perelman School of Medicine, University of Pennsylvania, 3710 Hamilton Walk, Philadelphia, PA (AMS).

## Abstract

Type 2 diabetes mellitus (T2DM) and hypertension are both associated with cognitive impairment and brain function abnormalities. We investigated whether abnormal cerebral blood flow (CBF) patterns exists in T2DM patients and possible relationships between aberrant CBF and cognitive performance. Furthermore, we examined the influence of hypertension on CBF alterations in T2DM patients.

T2DM patients (n = 38) and non-T2DM subjects (n = 40) were recruited from clinics, hospitals, and normal community health screenings. Cerebral blood flow images were collected and analyzed using arterial spin labeling perfusion functional magnetic resonance imaging (fMRI). Regions with major CBF differences between T2DM patients and non-T2DM controls were detected via 1-way ANOVA. The interaction effects between hypertension and T2DM for CBF alterations were also examined. Correlation analyses illustrated the association between CBF values and cognitive performance and between CBF and blood pressure.

Compared with non-T2DM controls, T2DM patients exhibited decreased CBF, primarily in the visual area and the default mode network (DMN); decreased CBF in these regions was correlated with cognitive performance. There was a significant interaction effect between hypertension and diabetes for CBF in the precuneus and the middle occipital gyrus. Additionally, blood pressure correlated negatively with CBF in T2DM patients.

T2DM patients exhibited reduced CBF in the visual area and DMN. Hypertension may facilitate a CBF decrease in the setting of diabetes. T2DM patients may benefit from blood pressure control to maintain their brain perfusion through CBF preservation.

## INTRODUCTION

Type 2 diabetes mellitus (T2DM) is a complex metabolic abnormality that increases the risk of cognitive decline.^1^ Approximately 3/4 of patients with T2DM live with hypertension.^2^ Interestingly, longitudinal and autopsy studies suggest that hypertension is a modifiable risk factor for the development and progression of cognitive decline.^3,4^ Therefore, hypertension is a likely risk factor for the cognitive impairment observed in T2DM patients. The precise relationship between blood pressure and brain function has not been elucidated in T2DM patients.

Research using cerebral perfusion, investigated via single-photon emission computed tomography, shows that diabetic patients exhibit decreased cerebral blood flow (CBF)^5–7^ and that CBF is directly correlated with cognitive performance.^8^ These studies included patients with Type 1 and Type 2 diabetes, and the sample sizes were limited. Arterial spin labeling (ASL) is a noninvasive functional magnetic resonance imaging (fMRI) technique^9–11^ that offers quantification of CBF. ASL perfusion fMRI has been applied in clinical settings; specifically, ASL has been used to detect early stages of dementia patients^12,13^ and has recently been used to assess CBF in T2DM patients. Findings have been mixed. Rusinek et al did not observe significant CBF changes between T2DM patients and healthy controls using ASL,^14^ whereas Tchistiakova et al observed that T2DM influenced cerebrovascular reactivity in relatively older individuals with hypertension.^15^ Regarding blood pressure, reductions in systolic blood pressure (SBP) in older individuals have been associated with increases in whole-brain gray matter CBF^16^ and hypertensive individuals displayed blunted CBF responses during completion of memory tasks.^17^ Research is needed to examine what regions exhibit altered CBF function in the T2DM disease state and to determine whether hypertension is involved in the fluctuation of CBF values in these regions.

Thus, we raise the hypothesis that T2DM patients would exhibit reduced CBF compared to non-T2DM controls and that reduced CBF in certain brain regions would correlate with deficits in specific cognitive domains; blood pressure would be inversely related with CBF in T2DM patients such that lower blood pressure would be linked with better perfusion condition. To address these issues, we analyzed CBF patterns to assess differences between T2DM patients and matched, non-T2DM controls using ASL perfusion functional MRI (fMRI) and observed the effect of hypertension on CBF changes as well as the associations between blood pressure and CBF alterations in T2DM patients.

## METHODS

### Subjects

Study protocol was approved by the Research Ethics Committee of the Affiliated Zhongda Hospital of Southeast University prior to study initiation. All participants provided written informed consent prior to any study procedures.

Seventy-eight subjects, including 38 patients with T2DM and 40 non-T2DM subjects, were recruited from the Affiliated Zhongda Hospital of Southeast University and community health screenings from June 2012 to September 2013. In order to participate, subjects had to meet the following inclusion criteria: right handed, educated for at least 6 years, and between 45 and 70 years of age. The T2DM diagnosis was based on the World Health Organization 1999 criteria.^18^ As described in our previous study,^19^ patients with retinopathy and peripheral neuropathy were excluded. Additionally, participants were excluded if they had a history of stroke, alcoholism, Parkinson's disease, head injury, epilepsy, major depression, other acute neurological or psychiatric illnesses, severe visual or hearing loss, anemia, thyroid dysfunction, cancer, severe heart diseases, and damaged liver/kidney function.

### Clinical and Neuropsychological Data Collection and Neuropsychological Tests

Demographic characteristics were collected at the time of MRI. Blood pressure was recorded as an average of 2 measurements taken after each participant had a 5-minute rest. Hypertension was defined as blood pressure ≥ 140/90 mm Hg or the use of antihypertensive medications. Following an overnight fast of at least 10 h, venous blood samples were collected at 8 A.M for all participants. Then each subject ingested a solution of glucose (75 g) in water over a few minutes, and the second blood sample was collected 2 h later (prior to the MRI scan). Fasting and postprandial blood glucose, HbA1c, and blood lipid (triglyceride, total cholesterol, low-density lipoprotein cholesterol, and high-density lipoprotein cholesterol) levels were assessed.

Each participant's cognitive function was assessed via the cognitive tests described in our previous study,^19^ including the Mini Mental State Exam (MMSE), Rey-Osterrieth Complex Figure Test (CFT), Auditory Verbal Learning Test (AVLT), Trail Making Test-A and B (TMT-A and TMT-B), and Clock Drawing Test (CDT), on the same day of the MRI scan in a quiet room. All tests were performed in a fixed order to evaluate general mental status, memory, attention, executive function, and visuospatial function. An experienced neuropsychiatrist facilitated this process, and a single-blinded method was used.

### MRI Acquisition

MR imaging was conducted in a 3.0T MRI scanner (Siemens MAGENETOM Trio) as previously described.^19^ In order to reduce head motion and scanner noise, cushions and earplugs were used. The subjects were instructed to relax and lie still in the scanner while keeping their eyes closed and avoiding either falling asleep or making sudden head motions, and to not think of anything in particular during the fMRI. Three-D T1-weighted images were acquired using the same parameters as in our previous study as follows:^19^ repetition time (TR) = 1900 ms, echo time (TE) = 2.48 ms, thickness = 1 mm, slices = 176, gap = 0 mm, flip angle (FA) = 90°, field of view (FOV) = 250 × 250 mm^2^, and matrix = 256 × 256. ASL images were obtained with a pulsed-ASL sequence (PICORE Q2T) using the parameters that are similar to those in previous study as follows:^20^ TR = 4000 ms, TE = 12 ms, FOV = 220 × 220 mm^2^, matrix = 64 × 64, slices thickness = 4 mm, gap = 1 mm, TI1 = 600 ms, and TI2 = 1600 ms. The entire scan time lasted 45 min, including the perfusion scan, anatomic scan, and other scans for blood oxygen level-dependent imaging and diffusion tensor imaging (data not reported here).

### Imaging Data Pre-Processing

All images were realigned to correct for head motion, and epochs of head movement greater than the width of 1 voxel were removed. The images were smoothed using a 3-dimensional, 6 mm FWHM Gaussian kernel. SPM8 (http://www.fil.ion.ucl.ac.uk/spm) and an in-house package developed at the University of Pennsylvania for automatic structural segmentation were used for image processing. All control and label images were realigned to the M0 image with the same spatial resolution from the same PASL acquisition. Perfusion images were then generated by pairwise subtraction and summation between the time-matched label and control images. Finally, quantitative CBF maps for all individuals were generated.^21^ These maps were then normalized to standard space using the Montreal Neurological Institute template brain. Global CBF was calculated, and the mean CBF images for each group were averaged.

We performed a voxel-based morphometry (VBM) approach to estimate gray matter (GM) brain volumes using the VBM8 toolbox (http://dbm.neuro.uni-jena.de/vbm) in SPM 8. During the pre-processing step of the VBM, DARTEL was used to improve the inter-subject registration of the structural images. Using a unified segmentation algorithm,^22^ the cerebral tissues were segmented into GM, white matter, and cerebrospinal fluid. Then, the T1 magnetic resonance images were normalized to the MNI template. Afterwards, the images were spatially smoothed with a 6 mm FWHM Gaussian kernel. Gray matter volumes (GMV) and white matter volumes (WMV) were generated during this process. Brain parenchyma volume (BPV) was calculated as the sum of GMV and WMV.

### Statistical Analysis

Demographic and neuropsychological data were analyzed using SPSS software and were 2-tailed, with the statistical significance level set at *P* < 0.05. All variables apart from MMSE were normally distributed. Differences between the T2DM patients and the non-T2DM controls were assessed using a *t*-test for normally distributed variables, a nonparametric Mann–Whitney *U* test for the asymmetrically distributed variable (MMSE), and a χ^2^-test for categorical variables. Bonferroni correction was used for multiple comparisons.

To examine between-group differences in brain volumes, a 1-way analysis of variance (ANOVA) was conducted with age, gender, and education level as nuisance covariates. Regions with CBF differences between groups were also detected via 1-way ANOVA in SPM8 with the same nuisance covariates. The threshold was set at *P* < 0.01 (cluster-level family-wise error (FWE) correction). A full-factorial model was utilized to examine potential interaction effects between hypertension and diabetes on CBF differences. The threshold was set at *P* < 0.05 (cluster-level FWE correction).

Based on previous studies,^23,24^ spherical regions of interests (ROIs) with a radius of 6 mm were defined in the left inferior parietal lobe (IPL) (MNI (Montreal Neurological Institute) coordinates *x* = −47, *y* = −57, *z* = 39), right IPL (MNI coordinates *x* = 49, *y* = −54, *z* = 39), right precuneus (MNI coordinates *x* = 1, *y* = −64, *z* = 43), and right occipital lobe (MNI coordinates *x* = 30, *y* = −81, *z* = 9). The left and right IPL were then combined into the bilateral IPL. For each subject, absolute CBF values in the ROIs were computed using the statistical parametric mapping Marsbar toolbox.^25^ Relative CBF values were calculated as the ratio of regional (absolute) CBF to global CBF. The relative CBF within these clusters was then plotted with the results from each neuropsychological test by partial correlation analysis. To investigate the relationship between CBF in certain brain regions and cognitive performance, we evaluated the partial correlation coefficients between the relative CBF in each respective ROI and each neuropsychological test. To investigate the relationship between CBF in certain brain regions and the severity of hypertension, we evaluated the partial correlation coefficients between the relative CBF in each respective ROI and blood pressure. All partial correlations were calculated after correcting for age, sex, and education. Thresholds were set at *P* < 0.05.

## RESULTS

Study demographics, clinical ratings, and cognitive performances are presented in Table 1 and Table 2. As expected, the T2DM patient group exhibited higher HbA1c, fasting and postprandial glucose levels (all *P* < 0.001) compared with the non-T2DM controls. Aside from these variables, the groups did not significantly differ on all other measures. In general, the T2DM patients performed worse than the non-T2DM controls regarding all neuropsychological tests (significant differences in CFT-copy, CFT-delay, DST, and TMT-B (*P* < 0.05)).

**TABLE 1:**
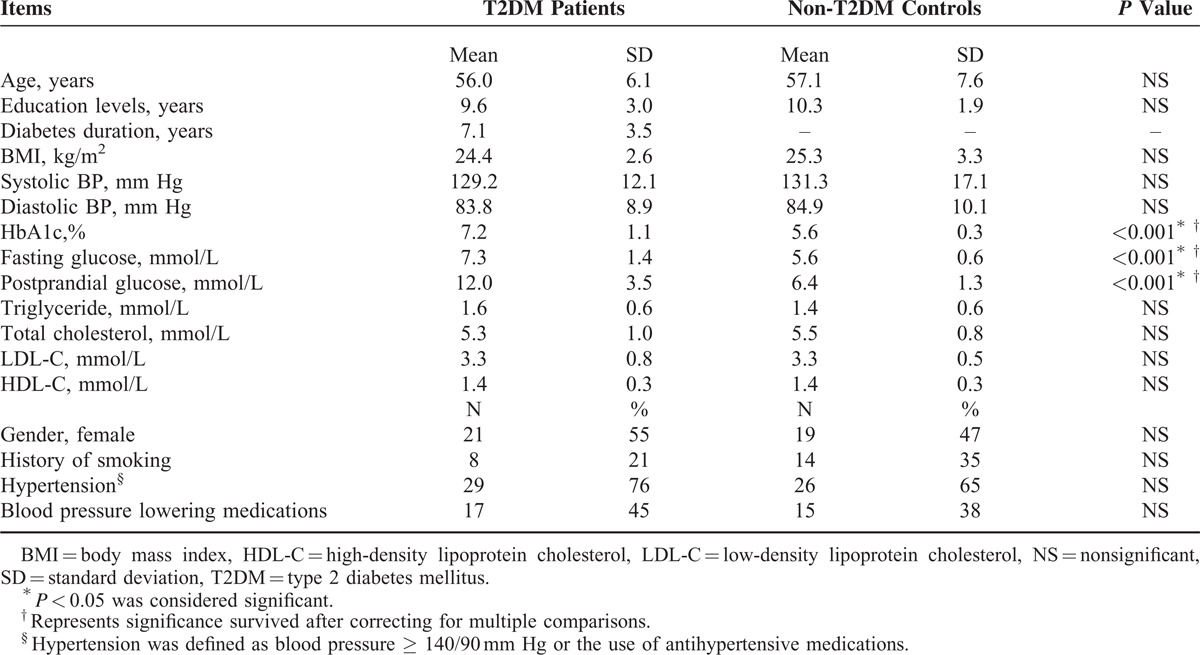
Demographic and Clinical Characteristics

**TABLE 2:**
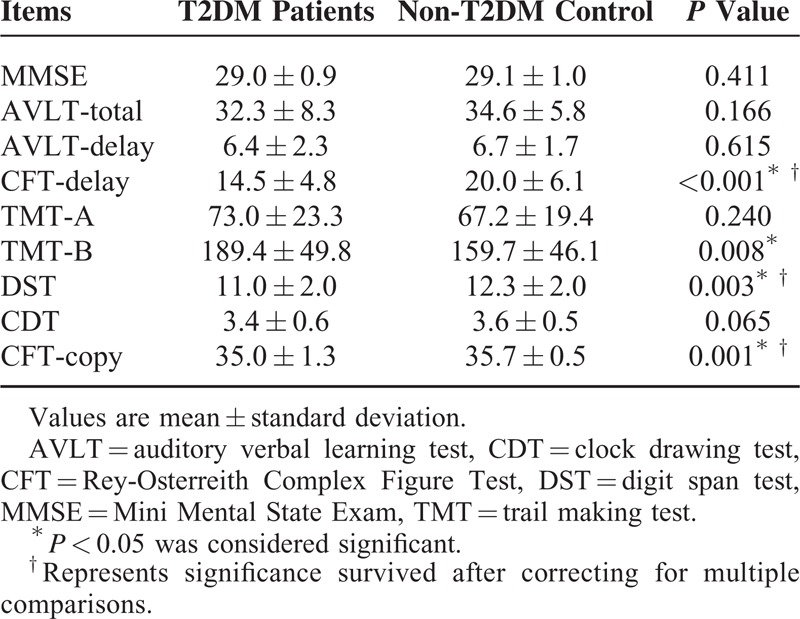
Cognitive Scores

**FIGURE 1:**
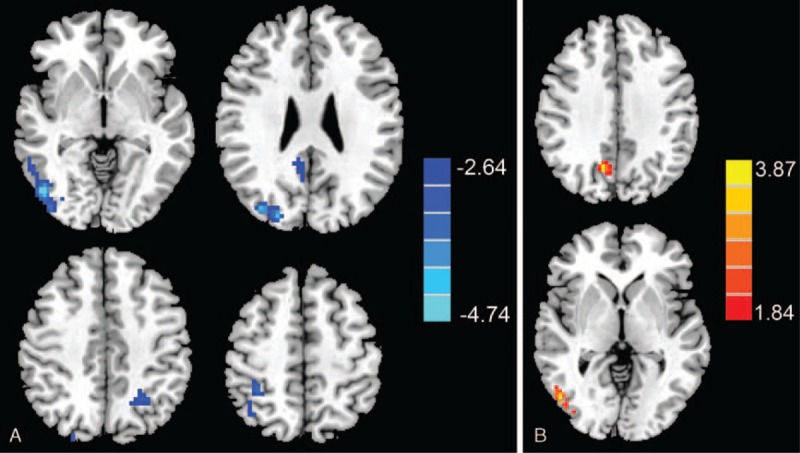
(A) Regions exhibiting significantly decreased CBF values. Compared with non-T2DM controls, T2DM patients exhibited decreased CBF primarily in visual and the default mode network regions, including the right middle occipital gyrus, bilateral inferior parietal lobe (IPL), and right precuneus. (B) Regions exhibiting an interaction effect between T2DM and hypertension on CBF values. The interaction effect between hypertension and diabetes was significant in the right middle occipital lobe and right precuneus.CBF = cerebral blood flow, IPL = inferior parietal lobe, T2DM = type 2 diabetes mellitus.

We did not observe any significant structural changes in specific brain regions (*P* > 0.05). Additionally, the GMV, WMV, and BPV of the 2 groups were not significantly different (Supplementary Table, which shows the comparisons of the brain volumes between groups). The T2DM patient group exhibited decreased CBF, primarily in the visual area and DMN, including the right middle occipital gyrus, bilateral IPL, and right precuneus (*P* < 0.01, corrected) (Table 3 and Figure 1A). The interaction effect between hypertension and diabetes was significant in the middle occipital lobe and the precuneus (Table 4 and Figure 1B).

**TABLE 3:**
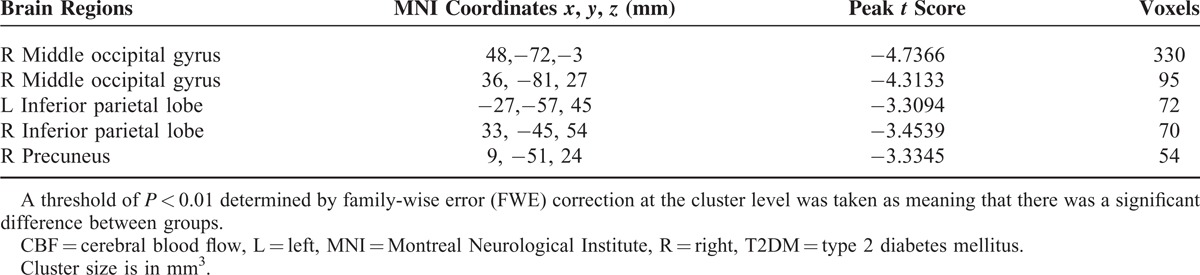
Regions Showing Significant Differences in CBF Between T2DM Patients and non-T2DM Controls

**TABLE 4:**
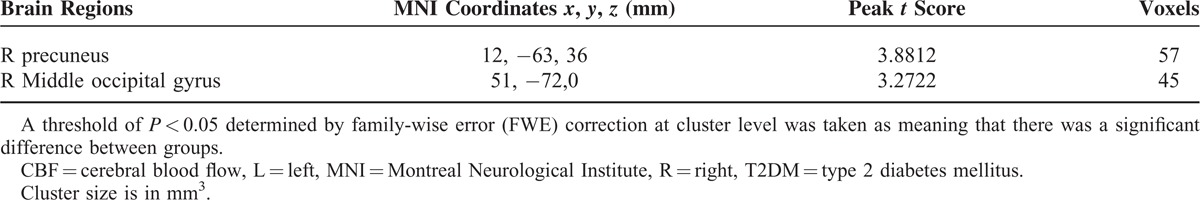
Regions Showing Interaction Effects Between Hypertension and Diabetes on CBF

**FIGURE 2:**
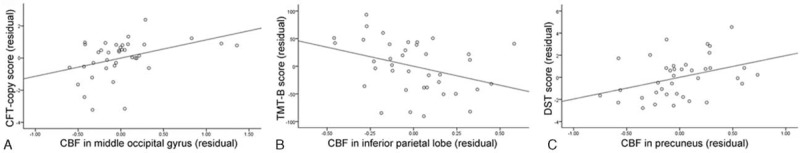
Correlation between cognitive performance and CBF values in T2DM patients. (A) Relative CBF in the middle occipital gyrus was significantly correlated with CFT-copy scores (*r* = 0.392, *P* = 0.020). (B) Relative CBF in the bilateral IPL was significantly correlated with TMT-B scores (*r* = −0.351, *P* = 0.039). TMT, Trail Making Test. (C) Relative CBF in the right precuneus was significantly correlated with DST scores (*r* = −0.371, *P* = 0.028). CBF = cerebral blood flow, CFT = Rey-Osterrieth Complex Figure Test, DST = digit span test, IPL = inferior parietal lobe, T2DM = type 2 diabetes mellitus, TMT = trail making test.

Correlations between CBF and cognitive performance are presented in Figure 2. Relative CBF in the middle occipital gyrus was associated with CFT-copy scores (*r* = 0.392, *P* = 0.020), relative CBF in the bilateral IPL was correlated with TMT-B scores (*r* = −0.351, *P* = 0.039), and relative CBF in the right precuneus correlated with DST scores (*r* = −0.371, *P* = 028) in T2DM patients. By calculating correlations between blood pressure and CBF values in the above 3 brain regions, we observed that the DBP of T2DM patients was inversely correlated with CBF in the middle occipital gyrus (*r* = −0.361, *P* = 0.033). Both DBP (*r* = −0.405, *P* = 0.016) and SBP (*r* = −0.343, *P* = 0.044) were inversely correlated with CBF in the precuneus of T2DM patients. We did not observe any significant correlations in non-T2DM subjects.

## DISCUSSION

The present study noninvasively quantified resting CBF in a large sample of T2DM patients. Compared to non-T2DM controls, T2DM patients exhibit reduced CBF, which was directly associated with cognitive performance. Interestingly, we also observed that hypertension exacerbated CBF reduction in T2DM patients, and poorly controlled blood pressure was associated with decreased CBF.

T2DM has been associated with structural brain changes,^26,27^ and we expected to observe brain atrophy in these patients. However, neither regional nor whole brain atrophy was observed in T2DM patients compared to matched controls. The most parsimonious explanation for this result is that the patients did not have any severe chronic comorbid complications and that every individual was in a very good general health condition. Research has shown that functional changes precede observable structural alterations in the setting of Alzheimer's disease^28^ and T2DM.^29^ An alternative possibility is that when compared to fMRI, VBM may be a less sensitive method for detecting subtle structural changes.

As a fundamental biological function, perfusion refers to the delivery of oxygen and nutrients to tissue via blood flow. Previous studies have observed abnormal perfusion in T2DM patients. Dandona et al reported CBF in diabetic patients for the first time, finding slightly increased CBF in these patients (however the difference was not significant); however, the authors did not provide information regarding the type of diabetes or the clinical characteristics of the diabetic patients.^30^ Rusinek et al also observed insignificant hypoperfusion in T2DM patients,^14^ which may have been due to the higher proportion of female patients in the T2DM group compared to the control group or that the ASL signal was acquired only at 2 axial locations. Tiehuis et al assessed relative global CBF by measuring the volume flow in the internal carotid arteries and basilar artery and observed that relative global CBF correlated with attention, executive function, and information processing speed in T2DM patients.^31^ However, these authors suggested that their results did not explain cognitive impairment in these patients because they also observed similar correlations in the control group. Brundel et al followed up the cohort and reported that cerebral hemodynamics did not play a major etiological role in either cognitive changes or brain alterations in T2DM patients.^32^ Both studies focused on global CBF rather than CBF in specific brain areas.

In our study, main effects of T2DM were primarily in visual and DMN regions. Consistent with our findings, Novak et al observed that T2DM patients exhibited reduced CBF velocity^33^ and exaggerated regional cerebral vasoreactivity to CO_2_ challenges in the parietal and occipital regions.^34^ The precuneus and parietal lobes are important components of the DMN, whereas the occipital lobe processes visual ability. When evaluating the cognitive profiles of the patients in this study, cognitive decline was associated with visuospatial and attention/executive function deficits. The precuneus is involved in visuospatial function and working memory, an important component of attention/executive function. Indeed, we found a significant correlation between CBF in the precuneus and DST, an assessment of working memory. We also observed significant correlations between CBF in the occipital lobe and performance on the CFT-copy as well as between CBF in the IPL and performance on the TMT-B, supporting the idea that there is a link between reduced CBF and deficits in both visuospatial and attention/executive function. In addition, because decreased CBF in the precuneus, posterior cingulate cortex (PCC), and lateral parietal cortex are consistently reported in Alzheimer's disease research,^35^ our data also illustrates that T2DM and Alzheimer's disease share similar brain pathophysiology. The involvement of these regions may represent a common pathophysiological process associated with cognitive decline in these diseases.

A region vulnerable to T2DM, the precuneus displays abnormal functional connectivity in T2DM patients in studies using various fMRI techniques.^29,36^ In patients with both T2DM and hypertension, perfusion was diminished in the occipito-parietal areas compared to patients with only hypertension.^18^ Additionally, hypertensive patients (without diabetes) exhibit reduced occipital brain perfusion.^37^ In healthy subjects, higher blood pressure has been correlated with lower glucose metabolism in the PCC-precuneus.^38^ Our data support and extend these findings by identifying a significant interaction between T2DM and hypertension for CBF in the middle occipital gyrus and precuneus, indicating that the existence of hypertension accelerates the decrease in CBF in the setting of diabetes, resulting in impaired visual-related cognition and attentional/ executive function.

Lower BP is associated with higher CBF in middle-aged hypertensive patients.^16,37^ Notably, hypertension exists in up to 3/4 of patients with T2DM.^2^ Increased blood pressure creates extra pressure load and results in hypertrophy of the medial layer of the major resistance vessels. The contraction of vascular smooth muscle causes a larger encroachment of the thick wall into the lumen, resulting in a steady increase in vascular resistance. During the course of hypertension, individuals tend to develop higher cholesterol, triglyceride, and plasma insulin levels, resulting in atherosclerosis.^39^ These changes lead to reduced blood flow, which may account for the lower CBF observed in patients with higher blood pressure. Based on our observation that CBF correlated with both DBP and SBP, T2DM patients may need to also maintain blood pressure control in order to achieve optimal brain function.

## LIMITATIONS

Some limitations of this study must be addressed. Due to the study's sample size, comprehensive changes in the brain and cognitive performance may not have been detected by our analysis. Second, this is a cross-sectional study and therefore cause and effect cannot be distinguished. It may be that T2DM reduced CBF in certain brain regions, or that T2DM damaged these brain regions, resulting in decreased metabolic demand. Hence, additional studies are needed to confirm these results in a longitudinal setting. Third, because hypertension is prevalent in middle-aged and older adults, we did not recruit exceedingly homogenous populations in either group. Although abnormal blood pressure in diabetic patients may be a confounding factor, no significant differences in either SBP or DBP were observed between the groups included in this study.

## CONCLUSION

This study provides evidence that patients with T2DM exhibit reduced CBF in visual and DMN regions and that decreased CBF in T2DM patients associated with poorer cognitive performance. Hypertension may exacerbate CBF reduction in T2DM patients, and blood pressure was positively associated with CBF levels. T2DM patients would likely benefit from blood pressure control in order to maintain brain perfusion via CBF preservation and prevent cognitive decline.

## Supplementary Material

Supplemental Digital Content
