# Supplementary material for: Blood Pressure is Associated With Cerebral Blood Flow Alterations in Patients With T2DM as Revealed by Perfusion Functional MRI
Source: Medicine (Baltimore). 2015 Dec 7;94(48):e2231. doi: 10.1097/MD.0000000000002231 (PMC4674216; doi:10.1097/MD.0000000000002231)
Supplement: Supplemental Digital Content [file medi-94-e2231-s001.docx]

**Supplemental Digital Content**

**Supplementary Table. Comparisons of the brain volumes between groups.**

| Brain volume | T2DM patients |  | Non-T2DM controls | *p*-value |
| --- | --- | --- | --- | --- |
| Gray matter | 760.9±68.2  756.6±59.6  1517.4±89..6 |  | 772.3±63.2  747.7±43.5  1520.0±80.2 | 0.443 |
| White matter |  |  |  | 0.452 |
| Brain parenchyma |  |  |  | 0.894 |

Values are mean ± standard deviation.
